# Quantitative Stain Mapping in X‐Ray Virtual Histology

**DOI:** 10.1002/advs.202519783

**Published:** 2026-01-20

**Authors:** Dominik John, David M. Paganin, Marie‐Christine Zdora, Lisa Marie Petzold, Patrick Ilg, Junan Chen, Sara Baggio, Johannes B. Thalhammer, Sami Wirtensohn, Julian Moosmann, Jörg U. Hammel, Felix Beckmann, Samantha J. Alloo, Jannis N. Ahlers, Madleen Busse, Julia Herzen, Kaye S. Morgan

**Affiliations:** ^1^ Research Group Biomedical Imaging Physics, Department of Physics, TUM School of Natural Sciences & Munich Institute of Biomedical Engineering Technical University of Munich Garching Germany; ^2^ School of Physics and Astronomy Monash University Victoria Australia; ^3^ Helmholtz‐Zentrum Hereon Institute of Materials Physics Geesthacht Germany; ^4^ Chair of Biomedical Physics, TUM School of Natural Sciences Technical University of Munich Garching Germany; ^5^ ImFusion GmbH Munich Germany; ^6^ Department Biological Safety German Federal Institute of Risk Assessment (BfR) Berlin Germany

**Keywords:** contrast agents, material decomposition, phase contrast, virtual histology, X‐ray imaging

## Abstract

Virtual histology using X‐ray micro‐computed tomography offers 3D tissue visualization, yet lacks the specificity of conventional histology, where targeted stains selectively highlight features like cell nuclei. While X‐ray‐compatible stains have now been developed, an unsolved challenge is the quantitative separation of their signal from the underlying tissue signal, which is essential for tissue‐specific imaging. This work presents the first method enabling 3D stain mapping on a histologically relevant scale. Applied to murine kidneys stained with different hematein‐lead complexes, the approach extracts molar contrast agent distributions alongside high‐contrast morphology at the micrometer scale and is successfully validated against K‐edge subtraction imaging. Moreover, the dual optical‐X‐ray properties of the stain enable direct spatial correspondence between X‐ray‐derived concentration maps and conventional optical histology of the same specimen, establishing a bridge between virtual and traditional histology. In summary, the proposed methodology provides tissue‐specific virtual histology with objective, quantitative metrics across millimeter to centimeter‐sized 3D volumes, opening pathways for immunospecific labeling and automated analysis of disease progression without physical sectioning.

## Graphical abstract

Virtual histology promises 3D tissue examination without physical sectioning, yet has lacked the tissue‐specificity of conventional pathology. This work demonstrates the first quantitative three‐dimensional stain mapping at histologically relevant resolution, separating contrast agent from tissue to reveal cellular features such as nuclei. The approach bridges virtual and traditional histology, enabling objective disease assessment across entire tissue volumes without destroying specimens.

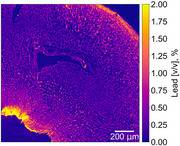

## 1 Introduction

Histopathology, the microscopic examination of diseased tissue, is the gold standard for diagnosing a multitude of diseases. However, traditional histopathology requires physically cutting tissue into thin slices for examination under an optical microscope, limiting analysis to 2D views that may show histological artifacts [1]. Though methods for reconstructing 3D information from conventional histological sections have been proposed [2], the process is labor‐intensive and requires advanced methods for registration due to artifact‐induced inconsistencies between slices [3]. To gain direct access to 3D information, methods like fluorescent light sheet microscopy [4, 5, 6] and X‐ray micro‐computed tomography [7, 8] are therefore active areas of research.

Virtual histology using X‐ray micro‐computed tomography offers a complementary approach to conventional histology by enabling 3D tissue examination without physical sectioning or the need for optical sample transparency, providing new insights into disease mechanisms and tissue architecture [9, 10, 11, 12, 13, 14, 15, 16, 17, 18]. The acquired 3D volume may also serve as a comprehensive overview of the sample structure, guiding subsequent targeted histological analysis of specific regions of interest [11, 19].

A major challenge for conventional X‐ray imaging of soft tissues is their naturally low contrast due to the subtle difference in density across different types of tissue. To address this limitation, various techniques have been developed that exploit not only how X‐rays are attenuated by tissue, but also how they are refracted or scattered. These phase‐contrast methods include propagation‐based imaging [20, 21, 22], grating‐based interferometry [23], crystal analyzer‐based methods [24], ptychography [25], single‐shot grid imaging [26, 27], and speckle‐based imaging [28, 29, 30]; the last two methods are more generally referred to as modulation‐based imaging [17, 31].

While all these methods significantly enhance tissue contrast and reveal those structural details that are difficult to access with X‐ray attenuation imaging alone, they are currently unable to provide the tissue specificity that conventional histopathology delivers. In optical microscopy, pathologists rely on a wide range of dyes that selectively highlight different cellular components such as nuclei, cytoplasm, or specific proteins. To bridge this gap, X‐ray‐compatible stains that target specific tissues and cellular structures have now been developed [32, 33, 34, 35, 36]. Notable examples include modified versions of hematein and eosin [34, 35], the two primary stains used in conventional pathology to highlight cell nuclei and cytoplasm, respectively. These X‐ray stains work by selectively altering the X‐ray interaction properties of targeted tissue components.

Since recorded X‐ray images always contain a combination of signal from both stain and tissue, the precise location and quantity of the stain in each voxel are not directly accessible. Material decomposition – a technique that separates materials based on their distinct X‐ray interaction properties [37, 38, 39, 40, 41, 42] – offers a potential solution. While a recent ptychography study successfully separated stain from tissue at the single‐cell level with sub‐micrometer resolution [42], extending this approach to the millimeter‐ to centimeter‐size scale required for histopathology applications has so far not been achieved.

For larger samples, modulation‐based X‐ray imaging offers a promising route [43, 44, 45, 46]. By analyzing sample‐induced distortions of a reference pattern imprinted onto the beam, algorithms such as Unified Modulated Pattern Analysis (UMPA) [47] can simultaneously retrieve X‐ray attenuation and electron number density, which should enable material decomposition. However, this has proven impossible in practice: second‐order phase effects originating at material boundaries create spurious edge artifacts [17], as current explicit modulator‐tracking methods only account for first‐order phase effects. These artifacts then dominate the attenuation signal under spatially coherent illumination. Since biological tissues consist predominantly of such interfaces, the measured attenuation coefficients bear little relation to actual material properties, precluding quantitative analysis. While correction methods have been proposed [40, 48, 49], a successful quantitative material decomposition has not been reported until now.

This paper presents the first method to retrieve quantitative stain concentrations alongside morphology at the micrometer scale required for pathology applications. The details of this novel modulation‐based X‐ray imaging method are provided in Section 4. In the body of the paper, we first demonstrate that modulation‐based imaging enables material decomposition in homogeneous material regions, confirming quantitatively accurate retrieval of attenuation coefficients and electron densities for materials with known X‐ray properties. This analysis also reveals the edge artifacts at material boundaries that have prevented quantitative analysis of biological tissues. We then introduce a novel physics‐informed correction that removes these edge artifacts, enabling accurate material decomposition of biological samples. Applied to mouse kidneys stained with a hematein‐lead complex [35] – a dual‐modal stain that creates contrast both in visible light (via hematein) and X‐rays (via lead) – material decomposition separates the stain and tissue signals, yielding three‐dimensional maps of lead concentration at micrometer resolution alongside high‐contrast morphology. Because phase‐contrast imaging inherently provides strong tissue contrast, cytoplasm‐specific staining [34] is not required to achieve histology‐like visualization. The quantitative accuracy is then validated against K‐edge subtraction imaging. Finally, we exploit the dual optical‐X‐ray properties of the stain to establish direct correspondence between X‐ray‐derived concentration maps and conventional optical histology of the same specimen, creating a direct bridge between virtual and traditional pathology.

## 2 Results and Discussion

### 2.1 Quantitative Material Decomposition

To test the limits of modulation‐based material decomposition in providing accurate electron density and attenuation coefficient measurements without edge‐artifact correction, and to calibrate the imaging system, a tomographic scan was performed on a phantom consisting of materials with known X‐ray properties. The phantom contained polyoxymethylene (POM), polyvinyl chloride (PVC), poly(methyl methacrylate) (PMMA) and polytetrafluoroethylene (PTFE), all submerged in pure ethanol. While ethanol, POM and PMMA cover the range of electron densities and attenuation values typically expected of soft tissue, PVC and PTFE span the higher values expected from lead‐based staining.

**FIGURE 1:**
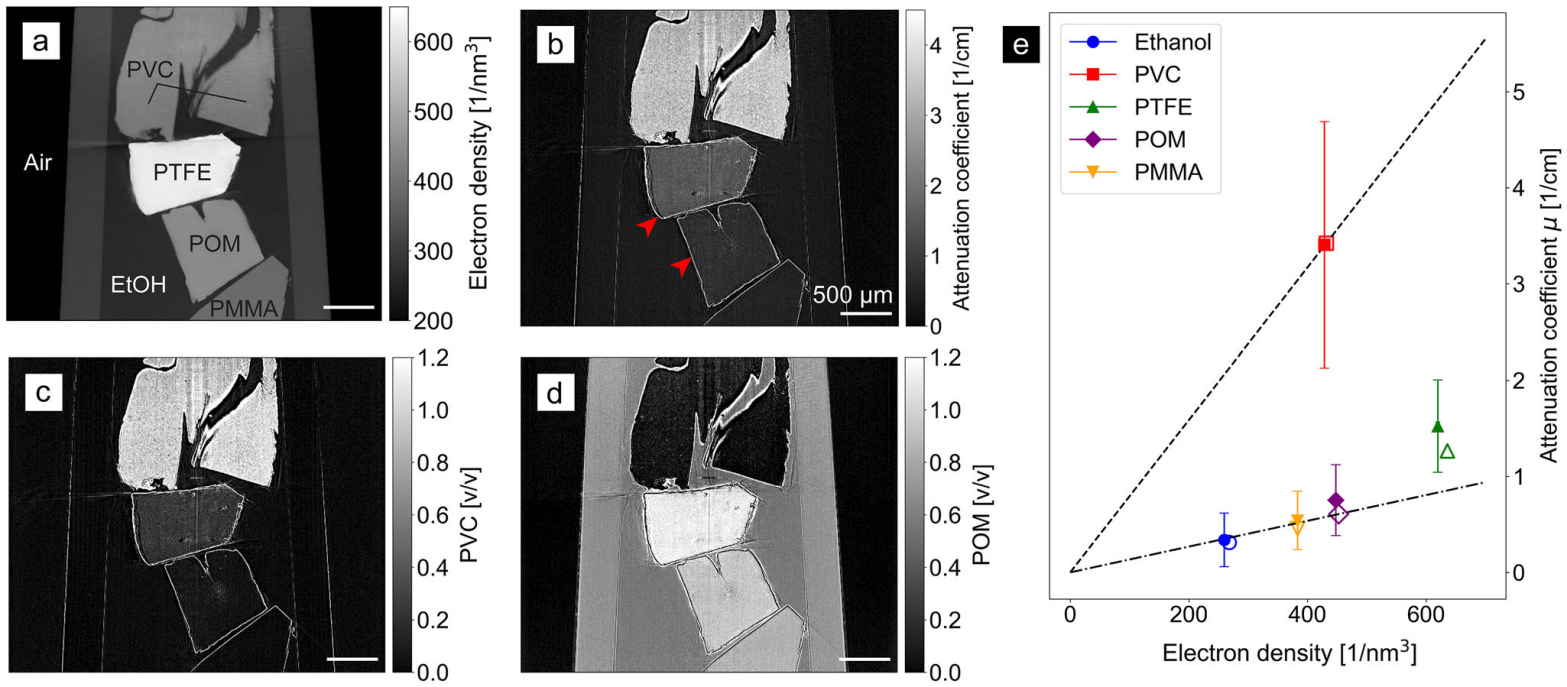
Central slice through tomographic reconstructions of the test sample. (a) Electron number density. (b) Linear attenuation coefficient, showing edge artifacts at material boundaries (red arrows). (c) and (d) Material decomposition into PVC and POM volume fractions, similarly showing edge artifacts. As expected, the volume fraction of the target materials is close to 1 within the corresponding part of the sample. (e) Measured electron densities and attenuation coefficients averaged over homogeneous regions (filled markers) show good agreement with theoretical values (open markers), except for a 2.3% deviation in PTFE electron density. The horizontal uncertainties are in the range of the measurement points. The dotted and dash‐dotted lines indicate the coordinate systems spanned by the theoretical values of PVC and POM, respectively, used for material decomposition. These lines serve as reference axes only and are not linear fits; the proximity of ethanol and POM to one of the axes is coincidental.

A slice through the sample is shown in Figure 1. The differences in material properties are clearly discernible in both the electron number density (a) and X‐ray attenuation coefficients (b). The averaged attenuation and electron number density values for each material, obtained as described in Section 4.4, are presented in panel (e). Nearly all values agree well with the theoretical predictions, demonstrating that the method accurately retrieves the physical quantities – consistent with previous work using a random diffuser as a modulator [50]. The one exception is the electron density of PTFE, which is 2.3% lower than expected at $(620\pm 3)\;{\mathrm{nm}}^{-3}$ compared to the theoretical value of $636\;{\mathrm{nm}}^{-3}$. This discrepancy may arise from variations in the actual material density or sample porosity relative to the theoretical bulk value.

The above values were obtained by averaging voxels within the interior of each material while excluding values near the edges. This exclusion was necessary because, as indicated by the red arrows in Figure 1b, the attenuation signal shows amplified values at the borders between materials. Since the materials are homogeneous and abrupt changes in attenuation are not expected, this demonstrates that modulation‐based tomography fails to properly extract attenuation coefficients near material boundaries. While this limitation is acceptable for large homogeneous blocks of material, it precludes quantitative analysis of biological specimens, where most of the sample volume consists of interfaces between different tissue types. In such cases, the majority of the attenuation signal would be spurious rather than reflective of actual material properties – this is the problem we address in Section 2.2 through our edge‐artifact correction.

Since modulation‐based imaging has not yet been used for material decomposition in the literature, we also demonstrate its material decomposition capabilities away from edges. For this purpose, a new coordinate system was defined based on the theoretical electron number density and attenuation coefficient values for POM and PVC, which were chosen because the properties of all materials in the sample fall within the span of this system. As shown in Figure 1c,d, the volume fraction of POM approaches 1 in the POM image and the fraction of PVC approaches 1 in the PVC image, which is the desired behavior. The other materials appear in different shades depending on their similarity to the base materials. Note that in this example, the volume fraction of PTFE exceeds 1; this is a mathematical consequence of its high electron number density, which causes it to be represented as an excess fraction relative to the base materials.

### 2.2 Quantitative Stain Mapping

To demonstrate the capabilities of our proposed virtual histology approach for separating stain and tissue signals, a modulation‐based tomography scan was performed on a mouse kidney stained with a hematein‐lead complex. As established in the previous section, the attenuation volume requires correction to remove edge enhancement effects that would otherwise produce negative intensity values and subsequently manifest as nonphysical negative stain concentrations. Below, we provide a brief intuitive explanation for the correction method, with a rigorous mathematical derivation presented in Section 4.3.

To obtain a transmission projection

(1) $$\tau \left(x,y\right)=\frac{I(x,y,z=0)}{I_{0}\left(x,y\right)},$$

where x and y are pixel coordinates in the image plane, we require both the intensity $I_{0}\left(x,y\right)$ without the object and the intensity I(x,y,z=0) immediately downstream of the object. However, because the detector is located at a propagation distance z=Δ from the sample, the measured intensity contains additional contributions proportional to the local refractive index decrement δ and linear attenuation coefficient μ. These effects are described by the transport‐of‐intensity equation [22, 51, 52]

(2) $$I\left(x,y,z=\Delta \right)=\left(1-\frac{\delta \Delta }{\mu }\nabla_{⊥}^{2}\right)I\left(x,y,z=0\right),$$

where $\nabla_{⊥}^{2}=\partial^{2}/\partial x^{2}+\partial^{2}/\partial y^{2}$ is the transverse Laplacian operator. This relationship shows that if the propagation distance and the local μ and δ values are known, the Laplacian phase effects can be reversed to recover a pure transmission image. We treat the stained tissue as a two‐material system consisting of soft tissue and lead, replacing the δ/μ ratio with the relative ratio $\left(\delta_{1}-\delta_{2}\right)/\left(\mu_{1}-\mu_{2}\right)$, where $\delta_{i}$ and $\mu_{i}$ denote the properties of each material [53, 54]. Average values for $\delta_{1}$ and $\mu_{1}$ are obtained by scanning an unstained section of the same kidney under identical conditions, while the well‐characterized properties of lead ($\delta_{2}$ and $\mu_{2}$) are taken from tabulated values [42]. Using these material properties, we apply a modified Paganin filter (detailed in Section 4.3) to the transmission projections. This correction removes the Laplacian phase effects that arise as coherent X‐rays propagate from the sample to the detector, thereby recovering the true intensity distribution at the sample exit plane.

**FIGURE 2:**
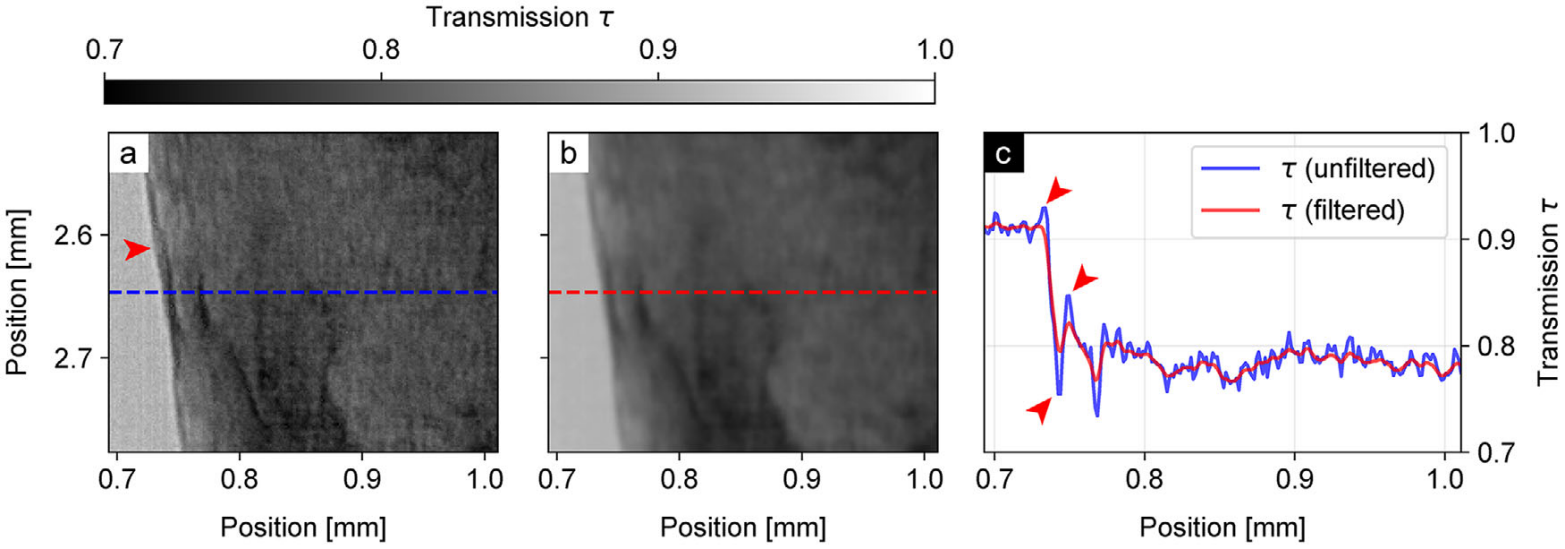
Comparison of cropped regions of (a) the unfiltered transmission image and (b) the filtered transmission image of the mouse kidney sample. The line plot (c) corresponds to the values across the blue and red lines in the images, averaged over two adjacent rows of pixels. The application of the filter results in a reduction of Laplacian phase effects and noise, most noticeable during the transition from the ethanol to the stained tissue (red arrows).

As shown in Figure 2 for a zoomed‐in transmission projection, this correction removes the amplified contrast at borders while improving the noise characteristics of the image. The difference is most visible at the ethanol‐tissue border (red arrow in panel (a)), which shows the expected direct transition from ethanol to tissue grey values only after filtering (red arrows in panel (c) indicate the uncorrected edge contrast).

While the filter may appear to degrade spatial resolution, a resolution analysis of the unfiltered and filtered attenuation volumes using Fourier ring correlation (see Section 4.7) reveals that the resolution actually improves slightly after filter application, from (7.36±0.13) μm to (6.71±0.04) μm (half‐bit). This effect likely stems from the suppression of high‐frequency noise and is consistent with previous literature showing that the filter term can simultaneously increase both the signal‐to‐noise ratio and the spatial resolution of images [55].

**FIGURE 3:**
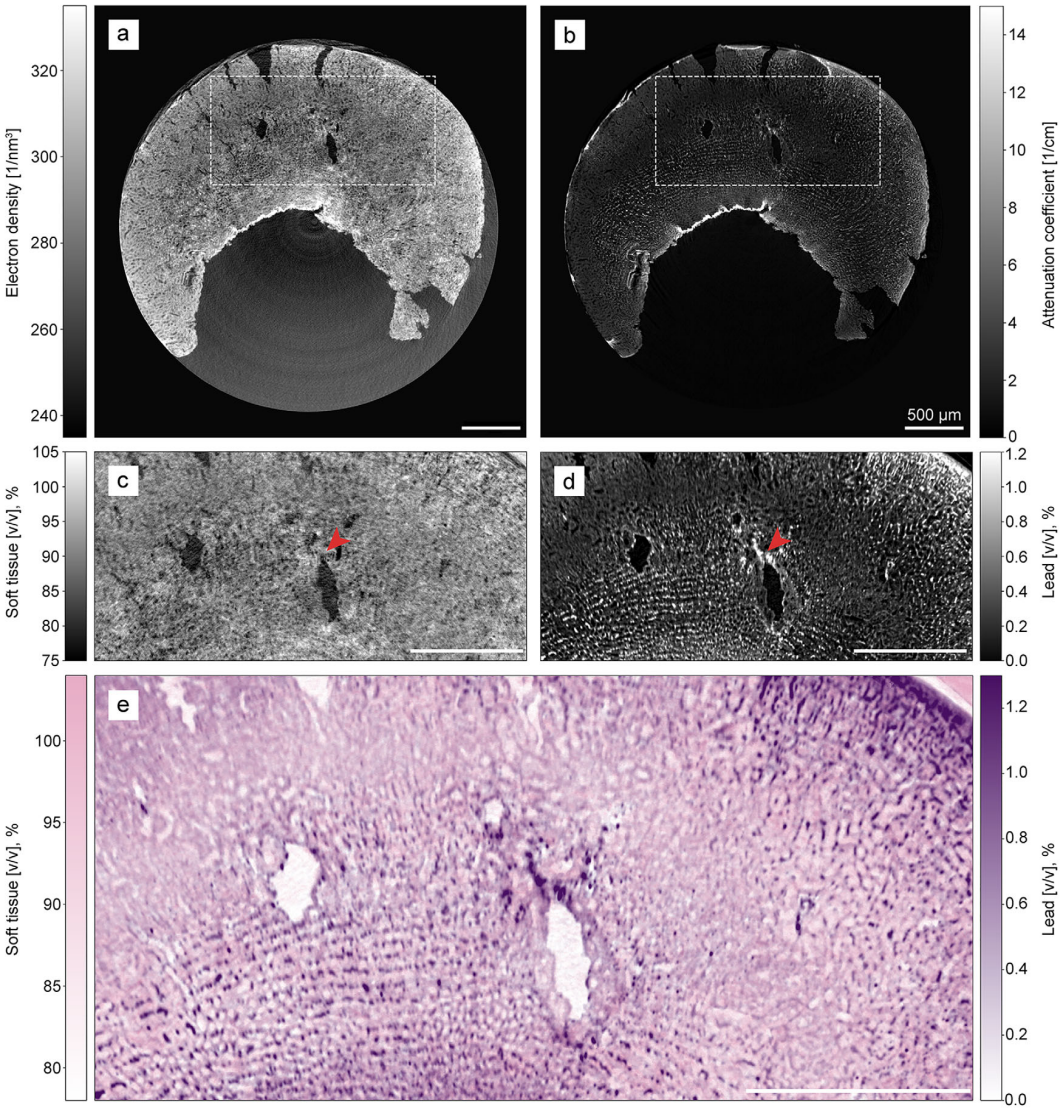
Slices through the tomographic reconstruction of the lead‐stained mouse kidney sample. (a) Electron number density. (b) Linear X‐ray attenuation coefficients (after removal of edge‐enhancement effects). (c) Soft tissue‐only image calculated based on data from the two regions of interest in the first row. (d) Lead volume fractions calculated analogously. (e) Merged soft tissue and lead volume fraction image. False colors are applied to mimic conventional histology images, where most cell structures appear pink due to eosin while cell nuclei are enhanced in purple. Red arrows indicate an example region showing a high electron number density in (a) mainly due to the presence of stain, which appears with reduced contrast in the corresponding soft tissue volume fraction image (c) since the staining contribution has been separated out into the lead volume fraction image (d). The sample container walls were removed using a mask in (a) and (b). Color mapping uses pink (#e75acc) for soft tissue (range [0.78, 1.04]) and purple (#480f62) for lead fraction (range [0.0, 0.013]), with intensity values scaled proportionally within these ranges to adjust color opacity from transparent to maximum saturation. The attenuation coefficient range of [$0\;cm^{-1}$, $14\;cm^{-1}$] corresponds to voxel optical densities in the range of [0, 0.002].

Tomographic reconstruction was then performed using the corrected attenuation projections and the original phase projections. Slices through the resulting electron number density and attenuation coefficient volumes are shown in Figure 3a,b. The attenuation coefficients can also be converted into unitless voxel optical densities to facilitate comparison with other works [56], mapping the attenuation coefficient range of [$0\;cm^{-1}$, $14\;cm^{-1}$] to voxel optical densities in the range of [0, 0.002]. Based on these signals, we apply material decomposition (see Section 4.5) to retrieve a soft‐tissue volume (c) and a lead volume (d), defined by the X‐ray interaction properties of unstained kidney and lead, respectively. This yields a three‐dimensional map of the lead volume fraction, providing access to the stain concentration in each voxel. The material decomposition also produces a soft tissue‐only image, which shows the sample morphology without the effects of the contrast agent. The complementarity of the two images is most visible in the area marked by the red arrows in Figure 3, which shows a strong signal in the lead image but not in the soft tissue image.

We then use a false‐color scheme inspired by conventional histology to overlay the lead concentrations (purple) on top of the soft tissue image (pink). In this scheme, the latter signal provides a backdrop of contrast by highlighting the general morphology, mimicking the role of the eosin stain in conventional histology. The superimposed lead map corresponds to the position of hematein, which is colored in violet. The resulting image is displayed in Figure 3e and demonstrates the selective highlighting by the stain, which is primarily taken up by cell nuclei. The modified hematein stain works by forming a positively charged hematein‐lead(II) complex in situ within acidified tissue that electrostatically binds to the negatively charged DNA phosphate backbone in cell nuclei [35]. The binding mechanism is the same as in traditional hematoxylin stains, but the lead's higher atomic number (Z=82) compared to aluminum (Z=13) or iron (Z=26) increases contrast for CT imaging. However, the high lead concentrations at the top right border of the tissue are likely not due to a strong presence of cell nuclei, but rather to excess stain that was not removed in the washing step.

The resolution was determined using Fourier ring correlation separately for both decomposed volumes (see Section 4.7), yielding consistent values of (7.2±0.4) μm (half‐bit criterion) and (8.3±0.6) μm (full‐bit criterion). These measurements are on the order of the size of mammalian nuclei of 5–10μm [57].

**FIGURE 4:**
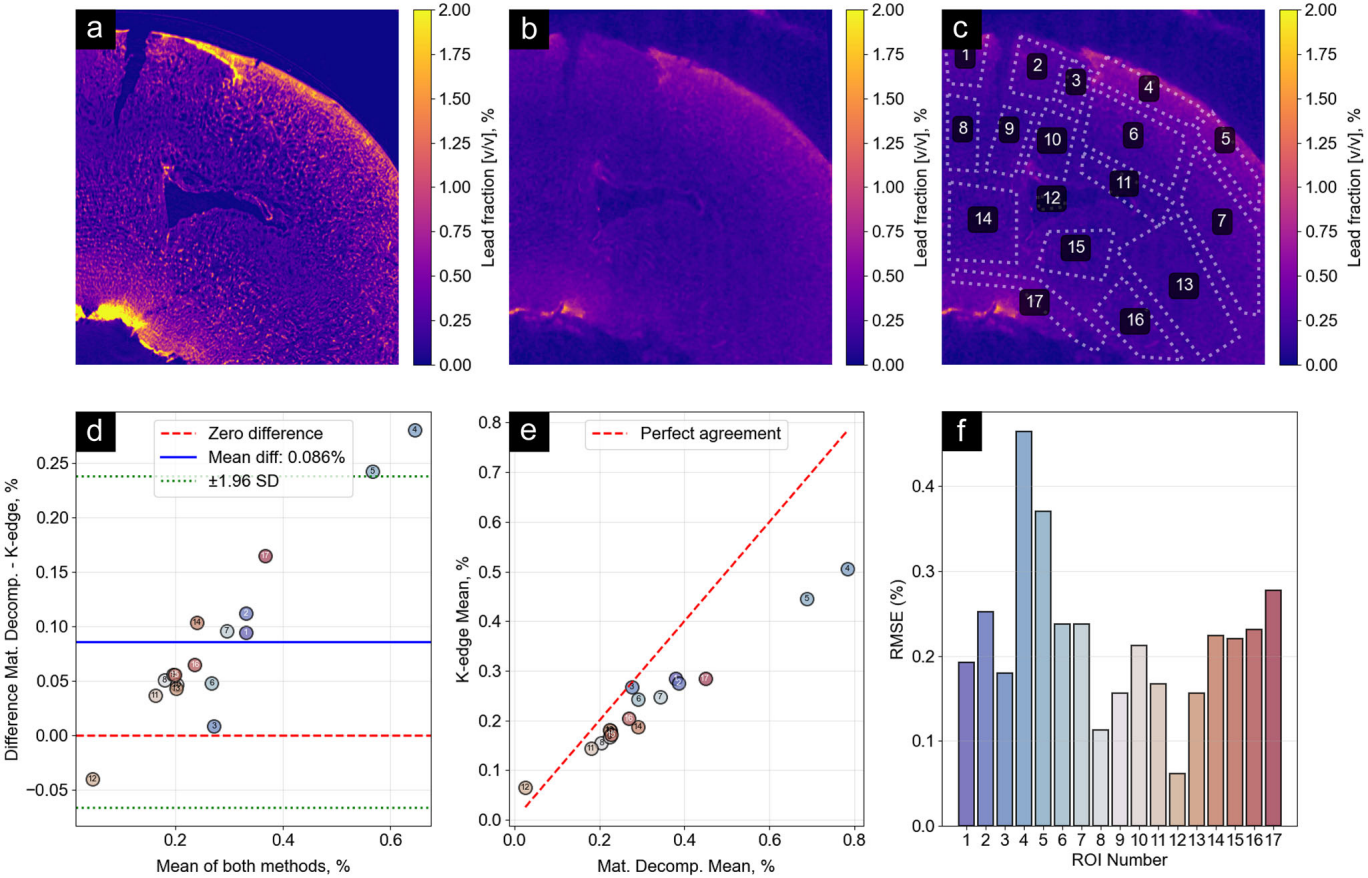
Comparison of lead concentrations obtained via material decomposition and K‐edge subtraction imaging. (a) Lead concentration from material decomposition. (b) K‐edge subtraction image of a similar slice through the sample. (c) Regions of interest (ROIs) used for quantitative comparison. (d) Bland–Altman plot showing good agreement between methods with a mean difference of 0.086 percentage points. (e) Scatter plot confirming good agreement, with exceptions in edge regions 4 and 5. The generally higher values from material decomposition and deviations at edges may be explained by stain leakage during the six‐month storage period between measurements, which would occur preferentially at tissue boundaries.

To confirm the quantitative accuracy of the lead concentrations retrieved using our approach, we compare the presented results to those acquired using the established method of K‐edge subtraction imaging (see Section 4.6). Figure 4 shows slices from the three‐dimensional concentration maps obtained using (a) the material decomposition approach and (b) the K‐edge approach. The slices are taken from similar locations in the kidney. Compared to the material decomposition approach in (a), the concentration map in (b) exhibits higher noise and lower spatial resolution at (27.5±1.5) μm (full‐bit) and (23.3±1.0) μm (half‐bit). This is due to the lower sample contrast and decreased beamline flux at the high energies on the order of 90keV required for this measurement. The quantitative agreement of both methods is analyzed by examining the regions of interest marked in (c) in both images. The region below ROI 17 was excluded from analysis, as the ethanol‐preserved specimen became brittle and this lower part detached during transport between the two measurements. A Bland–Altman analysis depicted in panel (d) indicates good agreement between the two methods. The mean difference between the two methods was 0.086 percentage points, with the material decomposition approach mostly reporting higher values than the K‐edge approach. The fresh ethanol used for the K‐edge measurement may have leached some stain. The scatter plot of the reported mean values for different ROIs in (e) as well as the root mean square error (RMSE) plot in (f) show good agreement with the exception of the edge regions 4 and 5, which may have been most susceptible to stain leaching. Means across the examined ROIs were highly correlated between methods (r=0.983, p<0.001).

Compared to K‐edge measurements, our material decomposition approach has the advantage of not requiring an energy switch during data acquisition. Another advantage is that tissue morphology is retrieved alongside stain concentrations. It is difficult to retrieve both with K‐edge imaging since the technique is typically performed at X‐ray energies above 50 keV (e.g., the K edges for iron, osmium, lead and bismuth are at 55.8, 73.8, 88.0, and 90.5 keV, respectively), and virtual histology to capture tissue morphology is performed at X‐ray energies below 25 keV to obtain sufficient contrast from these small pieces of biological soft tissue [10, 12, 14, 58, 59].

Furthermore, the phase and attenuation volumes obtained through our method are precisely spatially correlated since they originate from the same measurement, thus eliminating registration errors. In contrast, the two volumes measured above and below the K‐edge energy in conventional approaches may require spatial registration before analysis if sample drift or movement occurs, which can compromise quantitative accuracy.

### 2.3 Comparison to Conventional Histology

In a third experiment, a rat kidney was treated with a lower stain concentration than in Section 2.2 and embedded in paraffin wax. The stain concentration was chosen closer to values used in conventional histology protocols to enable subsequent optical microscopy analysis. A computed tomography scan was performed on the sample, and material decomposition was used to calculate a three‐dimensional stain concentration volume. After the scan, a histological section was obtained and converted into a greyscale optical density image.

**FIGURE 5:**
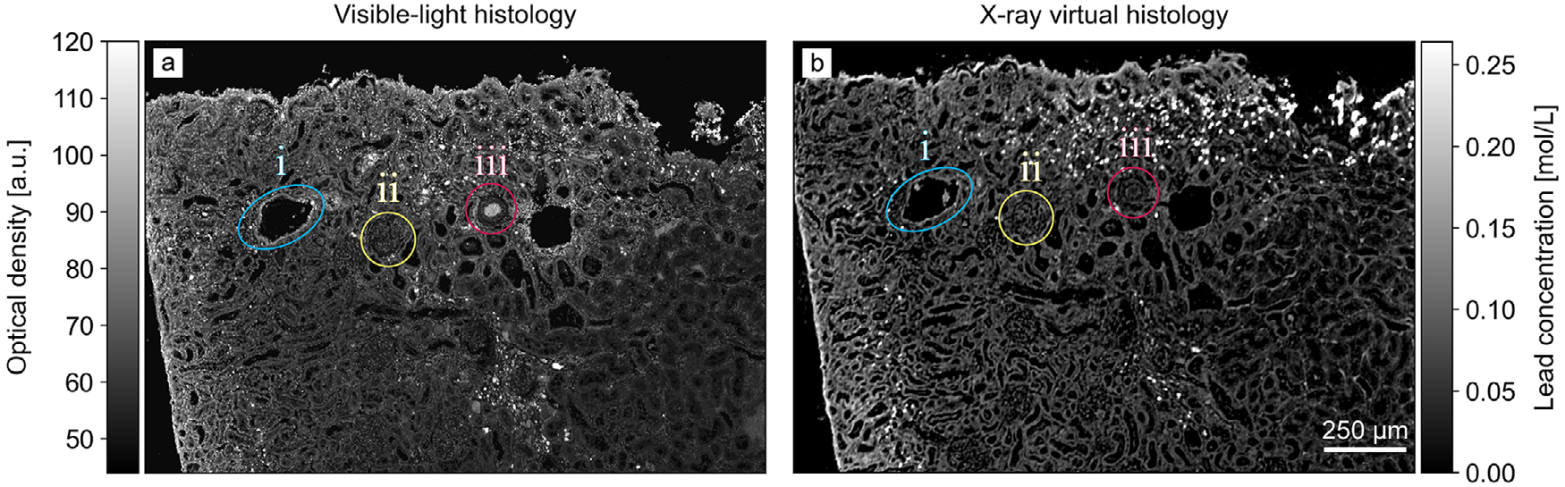
Visual comparison of (a) visible‐light microscopy image, converted to greyscale, with (b) a slice through the 3D concentration volume in a similar location. The similarity is explained by the dual optical‐X‐ray properties of the stain, allowing it to create contrast both in the visible light and X‐ray regime. While (a) allows distinguishing finer features better, (b) provides quantitative values for the stain concentration. Label (i) marks a renal vein, (ii) a glomerulus and (iii) a renal artery.

The examined X‐ray stain is a coordination complex that combines hematein, an optical dye, with lead ions. The stain, therefore, creates contrast in both the visible light and X‐ray regimes. This property is apparent in Figure 5, where the visible‐light histology image is compared to a similar slice in the 3D concentration volume. The slice through the volume was obtained using the 2D‐3D registration algorithm (see Section 4.7); note that perfect registration was not possible due to deformations from the physical cutting process.

The result shows very good visual agreement between the optical histology slice and the virtual concentration map, demonstrating the success of the material decomposition approach. While finer features are better resolved in visible‐light microscopy, the X‐ray virtual histology approach enables non‐destructive retrieval of stain concentrations in three dimensions. Using Fourier ring correlation, the spatial resolution in the axial plane of the X‐ray virtual histology dataset was determined to be (5.67±0.02) μm (half‐bit) and (6.42±0.01) μm (full‐bit).

The displayed image shows a region in the kidney cortex, which is of interest for diagnosing glomerular diseases, vascular pathologies, and tubular disorders. Anatomical features are well distinguishable in both images, including: (i) a renal vein branch, identifiable by its comparatively large lumen with thin walls; (ii) a glomerulus consisting of a capillary tuft enclosed within a Bowman's capsule; and (iii) a renal artery branch characterized by a thicker muscular vessel wall and intraluminal erythrocytes. Notably, the vessel lumen appears optically dense in (a) due to the inherent absorbing properties of erythrocytes rather than hematein staining. Conversely, in (b), the same lumen appears inconspicuous as erythrocytes lack nuclei and consequently do not take up the nuclear stain.

## 3 Conclusion and Outlook

In this work, we have developed an imaging approach that enables quantitative and comparable virtual histology of stained tissue with micrometer‐scale resolution. Our X‐ray modulation‐based method allows precise measurement of contrast agent concentrations alongside tissue morphology, providing objective metrics for tissue assessment that could reveal variations in agent distribution potentially linked to pathological changes.

We have validated our approach against conventional histology using an X‐ray stain that creates contrast in both the visible‐light and X‐ray regimes, creating a direct bridge from volumetric X‐ray data to conventional two‐dimensional histology. This approach may be useful for developing X‐ray stains and for 3D analysis of stained tissue samples without cutting.

Beyond standalone applications, our virtual histology approach complements conventional histology methods: the 3D X‐ray data provide a complete overview of the tissue sample, allowing researchers to identify regions of interest before physical sectioning. This guided approach can improve the efficiency of conventional histological analysis by ensuring that the most relevant areas are selected for high‐resolution optical microscopy.

While our method shows strong potential for the demonstrated applications, the following limitations should be addressed to broaden its range of applicability. First, achieving the same spatial resolution as conventional histology remains challenging for X‐ray microscopy. While the resolutions reported are on the order of mammalian cell nuclei size, this does not yet allow detailed inspection of individual nuclei, as is possible in conventional histology. While increased measurement time can improve spatial resolution, it is ultimately limited by the detector's point spread function (PSF). We note that initial work has been done to surpass this limit through a super‐resolution approach [60].

Second, further research is required into staining protocols to prevent high concentration gradients toward tissue edges, as observed in this study with samples approximately 5mm in their longest dimension. Addressing this edge effect will be essential before scaling the method to larger tissue specimens.

Third, our material decomposition assumes tissue consists of two basis materials – in this case, soft tissue and stain. While this proved accurate for the soft tissue samples studied here, as validated by agreement with K‐edge subtraction imaging (see Section 2.2) and a robustness test using tabulated values (see Section 4.5), the assumption becomes limiting for highly heterogeneous samples. Tissues containing materials with substantially different properties, such as bone minerals, would require at least a third modality, such as the dark‐field signal, for proper decomposition. Similarly, the proposed edge‐enhancement correction is designed for tissue systems that can be approximated as consisting of two materials and would thus also require modification.

Looking forward, several avenues for further research could extend this work. Our approach may be adopted for use with compact inverse‐Compton‐based sources [50], liquid‐metal‐jet sources [61], or conventional X‐ray sources [62]. Liquid‐metal‐jet sources may be especially suitable for transferring this protocol from synchrotron to laboratory settings, as they provide the high brilliance necessary to deliver the substantial doses required within practical acquisition times while achieving the spatial resolution and flux needed for this application. Alternative contrast agents should be explored, and incorporating the X‐ray dark‐field signal [30] could allow the use of nanoparticle‐based contrast agents in addition to conventional stains [63]. Our data may also serve as ground truth stain measurements for training AI‐based virtual staining methods, which attempt to replace physical staining with deep learning approaches that create color images based on native tissue [64, 65, 66]. The ability to generate large datasets of virtual histology images may also enable automated analysis using AI models for high‐throughput screening and quantitative assessment of tissue features.

## 4 Materials and Methods

### 4.1 Sample Preparation

For the experiments in Section 2.2, a wild‐type mouse kidney was extracted, fixed in formalin, and cut into six pieces, two of which were used for this study. The pieces were approximately $5\times 3\times 2\;mm^{3}$ in size. One piece was stained according to the hematein‐lead X‐ray stain protocol described in Ref. [35], while another piece was left in its native state. Hematein staining is a standard procedure in conventional histology, used to highlight cell nuclei in blue. The stain applied in this study is a modified version that uses lead acetate (rather than a lighter metal) as the intermediate binding partner between the negatively charged DNA backbone and hematein. This creates additional X‐ray contrast while retaining the binding properties of the original stain [35]. Immediately before the scanning, the samples underwent a dehydration process starting with a concentration of 70% (v/v) ethanol. The samples were finally scanned inside a sealed pipette tip containing 100% ethanol to prevent radiolysis‐induced bubbles that appear in aqueous solvents. A piece of PMMA was included in the pipette tip and acted as a calibration material for the phase measurements due to its known electron number density (see Section 4.4). The stained sample in Figure 2 and 4 was analyzed using K‐edge subtraction imaging at the Deutsches Elektronensynchrotron (DESY), Germany, approximately six months after the modulation‐based imaging at the Australian Synchrotron. Note that the sample was stored in the same solution for these 6 months, and then transferred into a new sample container filled with 100% ethanol.

The samples for the above experiments came from a female C57BL/6J mouse. Housing and dissection of organs was carried out at the neurophysiology section of the biological department, University of Hamburg, in accordance with the European Union's and local welfare guidelines (Behörde für Gesundheit und Verbraucherschutz, Hamburg, Germany; GZ G21305/591‐00.33).

For the experiments in Section 2.3, a piece of a rat kidney sample was prepared with a different protocol to achieve a more histologically compatible staining with lower concentrations, inspired by the method described by Metscher [67]. Two solutions were prepared: Solution A consisted of 66 mm lead(II) acetate trihydrate in distilled water (approximately 2.5g Pb(II) acetate in 10mL ${\mathrm{dH}}_{2}O$), while Solution B contained 1% hematein in 20% ethanol (1g hematoxylin, 0.2g sodium iodate, 20mL absolute ethanol, and 80mL ${\mathrm{dH}}_{2}O$, oxidized at room temperature for 24h without lid). Prior to staining, the sample was washed for 24h in distilled water to reduce precipitates according to the recommendations in Ref. [67]. Solutions A and B were then mixed in equal parts (1mL + 1mL), and the sample was transferred from the washing solution to the staining solution, where it remained for approximately 24h on a shaking plate. During this period, the sample was briefly removed for a laboratory‐based preview CT scan before being returned to the staining solution. Following staining, the sample was washed again in ${\mathrm{dH}}_{2}O$ to remove excess stain and stored for more than 24h until further processing. Finally, the sample underwent dehydration and embedding in paraffin for subsequent analysis. It was first scanned at the P05 beamline at PETRA III (see Section 4.2) and subsequently analyzed histologically. For this purpose, multiple slices of thickness 5μm were extracted using a microtome. The paraffin wax was removed using xylene, followed by incubation in a descending ethanol series. The slices were then briefly subjected to tap water and subsequently dehydrated using an ascending ethanol series back to xylene. Finally, the sections were mounted on glass slides with a coverslip.

The animal housing and organ removal for this sample were carried out at Helmholtz‐Zentrum Munich following the European Union guidelines 2010/63. The procedure was conducted in compliance with the ethical standards of the institution and approved by the responsible governmental body. Specifically, the experiments were performed under the license number ROB‐55.2‐2532.Vet_02‐21‐133.

### 4.2 Experimental Setup

**FIGURE 6:**
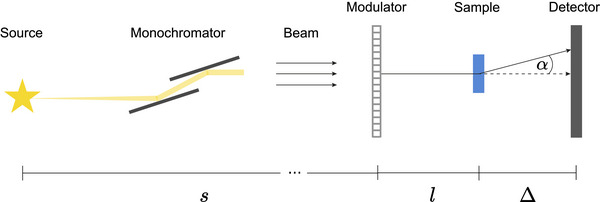
The X‐ray beam originates from a bending magnet source (MCT) or an undulator (P05) and is set to the desired energy using a double multi‐layer monochromator. After a distance s, the beam impinges upon the modulator, which introduces a phase shift in regular intervals across the wavefront. The sample is positioned at a distance l from the modulator, and the detector is at a distance Δ from the sample.

The material decomposition experiment described in Section 2.2 was performed at the microCT beamline (MCT) of the Australian Synchrotron, a third‐generation light source operating with an electron energy of 3GeV and a beam current of 200mA in the storage ring [68]. A sketch of the setup is shown in Figure 6: the radiation originates from a 1.3T bending magnet and is then filtered to an energy of 25keV using a double‐multilayer monochromator. After a distance s=21.3m, the beam impinges on a Talbot array illuminator [46] made of silicon with a period of 10μm and a design height of 25.7μm, which we refer to as the modulator. This height corresponds to introducing a 2π/3 phase shift at the design energy of 30keV. The sample was placed l=0.19m downstream of the modulator and was followed by the sCMOS detector placed a further 0.16m downstream. For the camera, a 4.5× magnification lens was used, leading to an effective pixel size of 1.44μm at the detector position and a field of view of 3.7× $3.1\;mm^{2}$ (width × height).

For each scan, 9 consecutive tomographic acquisitions were performed with an exposure time of 40ms per projection. For each tomography, the modulator was placed in a different transverse position. The signal retrieval was performed using the local feature‐tracking algorithm UMPA [47], specifically a runtime‐optimized version [69] with a window size parameter of 1 (effective window size 3×3 pixels). The electron number density and attenuation volumes were retrieved from the UMPA‐processed projections with the filtered backprojection algorithm of the *Core Imaging Library (CIL)* [70], which uses the *ASTRA Toolbox* [71] as its backend.

The calibration sample was scanned using 1800 projections acquired over an angular range of $180^{\circ }$. Because the biological samples were too large to fit the detector's horizontal field of view, they were scanned with an offset rotation axis in 3600 projections over an angular range of 360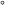 (see Section 4.8).

Additionally, the lead‐stained mouse kidney sample was measured at the High Energy Materials Science Beamline P07, which is operated by Helmholtz‐Zentrum Hereon at PETRA III, DESY in Hamburg, Germany [72]. The measurement was conducted at this beamline because the K‐edge energy of lead (88keV) falls outside the operating specifications of the MCT beamline. The P07 beamline enabled two consecutive absorption scans at a propagation distance of 3cm and energies of 87keV and 89keV, respectively. The detector has an array of 7920×6004 pixels with a pitch of 4.6μm, which leads to an effective pixel size of 0.90μm using 5.1‐fold magnification. 5000 projections were acquired over 180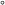 for each scan with an exposure time of 350ms each. The sample fitted into a single field‐of‐view, meaning no stitching was required. To correct for a slight deformation of the sample during the scan, the volumes captured at two energies were deformably registered with *ImFusion* software before quantitative analysis [73]. Additionally, both volumes were bilaterally filtered individually to reduce the impact of noise while retaining edge sharpness. Before volumetric reconstruction analogously to the previous experiment, the data was binned fivefold to reduce the impact of noise.

The material decomposition experiment described in Section 2.3 was conducted at the Imaging Beamline P05, which is operated by Helmholtz‐Zentrum Hereon at PETRA III, DESY in Hamburg, Germany [74, 75]. An undulator source was used in combination with a double crystal monochromator to create a highly coherent and monochromatic beam with an energy of 20keV. The detector uses a CMOSIS CMV 20000 sensor with 5120×3840 pixels and a pitch of 6.5μm [76], leading to an effective pixel of 1.28μm with the fivefold magnification objective. The experimental setup is similar to the setup at the microCT beamline used for Section 2.2. The modulator was a Talbot array illuminator with a period of 7μm and a design height that introduces a 2π/3 phase shift at its design energy of 20keV. Both the distances l and Δ were equal to 0.175m. 3001 projections with an exposure time of 80ms each were acquired over an angular range of 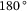 for each of the 16 modulator positions. A dynamic reference correction was performed by decomposing the recorded reference images into eigencomponents [77]. Each projection was then corrected with the linear combination of eigencomponents that yielded the best least‐squares fit in a sample‐free region, as described in Ref. [78]. The signal retrieval was then performed using UMPA [47] with a window size parameter of 1 (effective window size 3×3 pixels). The tomographic reconstruction was performed using the Feldkamp‐Davis‐Kress algorithm implemented in the *Xaid* software suite (*Mitos GmbH*, Garching b. München, Germany).

### 4.3 Accurate Retrieval of X‐Ray Attenuation in Two‐Material Mixtures

In conventional X‐ray imaging, the 2D transmission image τ(x,y) represents the logarithmic ratio of transmitted intensity I(x,y) to incident X‐ray intensity $I_{0}\left(x,y\right)$, where (x,y) are real‐space coordinates in the image plane. According to the Beer‐Lambert law, the transmission is directly related to the sample's linear X‐ray attenuation μ(x,y,z) coefficient via

(3) $$\tau \left(x,y\right)=-\ln\left(\frac{I(x,y)}{I_{0}\left(x,y\right)}\right)=\int_{0}^{T(x,y)}\mu \left(x,y,z\right)\;dz,$$

where T(x,y) is the exit surface of the sample and z refers to the coordinate along the beam propagation direction. When imaging samples with coherent X‐ray sources and a non‐negligible sample‐to‐detector distance, however, the image retrieved by application of Equation (3) contains additional contributions related to the Laplacian of the sample‐induced X‐ray wavefield phase shift Δϕ(x,y) (cf. Equation (2)). These manifest as increased contrast at the material boundaries. While this increased contrast benefits methods such as propagation‐based imaging [22, 55] or implicit speckle‐tracking [79], it is problematic for accurate material decomposition if left unaccounted for, since material decomposition assumes a clear separation between the phase and attenuation signal (see Section 4.5). Although such Laplacian phase effects can be simulated and removed [40], this method requires manual tuning of parameters, and any mismatch can introduce noise. In this section, we therefore propose an alternative approach that takes into account the Laplacian phase effects to obtain an image solely related to the sample's attenuating properties.

The Paganin filter, as first proposed by Paganin et al. [22], is a method for optics‐free phase‐retrieval using a single propagation distance and the assumption that the sample consists of a single material. The strength of the filter depends on the δ/μ‐ratio of the material, where δ is its refractive index decrement and μ is its linear attenuation coefficient. It has been shown that the method can be extended to multiple‐material samples by considering a piece of material 1 embedded in a different material 2 [53, 54]. The δ/μ‐ratio is then replaced by a relative ratio

(4) $${\left(\frac{\delta }{\mu }\right)}_{\text{rel}}=\frac{\delta_{1}-\delta_{2}}{\mu_{1}-\mu_{2}},$$

where $\delta_{i}$ and $\mu_{i}$ refer to the refractive index decrement and linear attenuation coefficient of each material [53, 54]. We extend this approach to arbitrary mixtures of materials by first assuming that each voxel of the reconstructed volume consists of a mixture of two materials, a fraction f of material 1 and a fraction (1−f) of material 2. The effective δ and μ of a given voxel are then the linear combinations

(5) $$\delta_{\text{mix}}=f\;\delta_{1}+\left(1-f\right)\;\delta_{2}$$

and

(6) $$\mu_{\text{mix}}=f\;\mu_{1}+\left(1-f\right)\;\mu_{2}$$

(cf. Ref. [80]). Assuming such a mixture with a fraction $f_{1}$ is embedded within a second mixture of fraction $f_{2}$, the relative δ/μ ratio using Equation (4) is

(7) $$\begin{array}{cc} {\left(\frac{\delta }{\mu }\right)}_{\text{rel}} & =\frac{\left[f_{1}\delta_{1}+\left(1-f_{1}\right)\delta_{2}\right]-\left[f_{2}\delta_{1}+\left(1-f_{2}\right)\delta_{2}\right]}{\left[f_{1}\mu_{1}+\left(1-f_{1}\right)\mu_{2}\right]-\left[f_{2}\mu_{1}+\left(1-f_{2}\right)\mu_{2}\right]}\\ & =\frac{\left(f_{1}-f_{2}\right)\left(\delta_{1}-\delta_{2}\right)}{\left(f_{1}-f_{2}\right)\left(\mu_{1}-\mu_{2}\right)}=\frac{\delta_{1}-\delta_{2}}{\mu_{1}-\mu_{2}}. \end{array}$$

The above equation shows that knowledge of the mixtures $f_{1}$ or $f_{2}$ is not required via this approach. Thus, the same Paganin filter is valid for arbitrary mixtures of two given materials.

For the case of single‐distance propagation‐based imaging, Croton et al. have demonstrated how a pure attenuation image of a sample may be obtained by applying a suitably modified Paganin filter to the recorded intensity images [81]. This is achieved by assuming the object is made of two different materials, and the total projected thickness of the object varies slowly. This is valid for the samples examined in this work due to the cylindrical, ethanol‐filled containers used as sample tubes.

We extend the approach by Croton et al. to modulation‐based imaging, where a sample is imaged at multiple different modulator positions. An approximate uncorrected transmission image may be obtained by averaging over all sample images $I_{i}\left(x,y\right)$ and reference $I_{0,i}\left(x,y\right)$ images:

(8) $$\tau \left(x,y\right)=\sum_{i}\frac{I_{i}\left(x,y\right)}{I_{0,i}\left(x,y\right)}.$$

This approximation assumes that by superimposing a sufficient number of different modulator positions, the effect of the mask on the image cancels out on average. The filter is then applied to this image (cf. Refs. [81, 82]) to obtain the corrected transmission image

(9) $$\tau^{\prime }\left(x,y\right)=F^{-1}\left[\frac{F\left[\tau (x,y)\right]}{1+{\left(\frac{\delta }{\mu }\right)}_{\text{rel}}\;\Delta \;k_{⊥}^{2}}\right],$$

where Δ is the distance between the sample and the detector. Here, F denotes Fourier transformation with respect to x and y and $F^{-1}$ is the corresponding inverse Fourier transformation; $k_{⊥}=\left(k_{x},k_{y}\right)$ denotes the Fourier‐space coordinates corresponding to (x,y). The resulting expression is similar to a previous result derived in the context of single‐shot speckle‐based imaging [82].

The resulting projection is thus corrected for propagation‐based effects related to the Laplacian of the X‐ray wavefield phase and accurately quantifies the transmission of X‐rays through the sample; additionally, the amount of noise in the image is reduced [55]. The filter term is derived based on Equation (2), namely the finite‐difference approximation of the transport‐of‐intensity equation [51], given by Refs. [22, 52] as

(10) $$I\left(x,y,z=\Delta \right)=\left(1-\frac{\delta \Delta }{\mu }\nabla_{⊥}^{2}\right)I\left(x,y,z=0\right).$$

Here, $\nabla_{⊥}$ refers to the nabla operator in the x‐y‐plane, meaning $\nabla_{⊥}^{2}=\partial^{2}/\partial x^{2}+\partial^{2}/\partial y^{2}$ is the transverse Laplacian. This formulation conserves the total intensity in the imaging plane Ω (assuming vanishing flux across its boundary region ∂Ω) due to the divergence theorem

(11) $$-\frac{\delta \Delta }{\mu }{\;\int \int \;}_{\Omega }\nabla_{⊥}\cdot \nabla_{⊥}I\left(x,y,z=0\right)\;dx\;dy=0.$$

The transmission projections used to obtain the X‐ray attenuation coefficient volume in Figure 3 were corrected with a relative δ/μ‐ratio based on the X‐ray interaction properties of soft tissue and lead. An estimate for the soft tissue was obtained by averaging the electron number density $\rho_{e}$ and linear X‐ray attenuation coefficients μ in an unstained piece of mouse kidney over a rectangular region of 100 consecutive slices; the values measured are $\rho_{\text{e,1}}=299\;{\mathrm{nm}}^{-3}$ and $\mu_{1}=0.567\;cm^{-1}$. The attenuation coefficient at an energy of 25keV and the electron number density of atomic lead were obtained using a look‐up table [83] and are $\mu_{2}=550\;cm^{-1}$ and $\rho_{\text{e,2}}=2703\;{\mathrm{nm}}^{-3}$, respectively.

### 4.4 Retrieval of Electron Number Densities

The electron number density $\rho_{e}$ of the sample can be retrieved using X‐ray phase measurements. UMPA tracks sub‐pixel displacements $\left(u_{x},u_{y}\right)$ of the reference pattern as a result of phase effects [47]. These displacements across the detector face in pixels can then be converted into refractive angles using

(12) $$\left(\phi_{x},\phi_{y}\right)=\frac{2\pi }{\lambda }\;\left(u_{x},u_{y}\right)\frac{p}{\Delta },$$

where $\left(\phi_{x},\phi_{y}\right)$ are the x‐ and y‐derivatives of the object's phase ϕ(x,y), λ is the wavelength, and p is the pixel size at the detector face; note that the displacements $\left(u_{x},u_{y}\right)$ are dimensionless because they have units of pixels [47]. The phase of the object can then be obtained by 2D Fourier integration of the two differential phase signals [84, 85, 86]:

(13) $$\phi \left(x,y\right)=F^{-1}\left(\frac{F(\phi_{x}+i\phi_{y})}{ik_{x}-k_{y}}\right).$$

It shall be noted that the Fourier space coordinates use the effective pixel size in the image plane (instead of at the detector face, hence now including magnification effects), although the distinction is not important if the calibration at the end of this section is performed. We define the phase of the air surrounding the object to be 0 by subtracting the average phase value in an air‐filled region of interest.

The projected electron number density may be calculated using the recovered phase via

(14) $$\rho_{e,⊥}\left(x,y\right)=\frac{-\phi (x,y)}{\lambda \;r_{e}},$$

where $r_{e}$ is the classical electron radius [52]. Finally, the electron number density in each voxel is obtained by tomographic reconstruction of these projections.

To minimize systematic errors in the obtained electron number density, we make use of a calibration procedure employed in Ref. [50]. Here, a reference material is used to rescale the final position‐dependent electron number density according to

(15) $$\rho_{e,\text{cal}}\left(x,y,z\right)=\frac{\rho_{e,\text{raw}}\left(x,y,z\right)-{\bar{\rho }}_{e,\text{bg}}}{{\bar{\rho }}_{e,\text{ref}}-{\bar{\rho }}_{e,\text{bg}}}\;\rho_{e,\text{theo}},$$

where $\rho_{e,\text{cal}}\left(x,y,z\right)$ represents the three‐dimensional calibrated electron number density map, $\rho_{e,\text{raw}}\left(x,y,z\right)$ is the electron number density map before calibration, ${\bar{\rho }}_{e,\text{bg}}$ and ${\bar{\rho }}_{e,\text{ref}}$ are the mean electron number density of the air background and reference material, respectively, and $\rho_{e,\text{theo}}$ is the theoretical electron number density of the reference material. In this work, we used a piece of poly(methyl methacrylate) (PMMA) as a reference material. Its theoretical electron number density is $383\;{\mathrm{nm}}^{-3}$, which was calculated using the *xraylib* library [83]. Prior to analysis, all electron number density images were rescaled using Equation (15), setting the measured PMMA electron number density value to the theoretically expected value. The exception is the rat kidney sample from Section 2.3, which was calibrated using its surrounding paraffin block as a reference material. The electron number density of the specific paraffin used in the experiment ($\rho_{e,\mathrm{paraffin}}=299\;{\mathrm{nm}}^{-3}$) was known due to a computed tomography experiment for calibration that included a PMMA rod and paraffin.

### 4.5 Material Decomposition

Material decomposition aims to determine the composition of heterogeneous samples by expressing value pairs of complementary measurements in terms of known reference materials. The method makes use of the fact that any material with a linear attenuation coefficient μ and electron number density $\rho_{e}$ may be mathematically described as a linear combination of two base materials with attenuation coefficients and electron densities $\mu_{i}$ and $\rho_{e,i}$, respectively [80]. The volume fractions $v_{i}$ represent the relative amount of each base material contained in a voxel that reproduces both the measured attenuation coefficient and electron number density. In order to obtain these volume fractions of the chosen base materials, a change of the basis of the coordinate system needs to be performed [42]:

(16) $$\left(\begin{array}{c} \mu \\ \rho_{e} \end{array}\right)=A\;\left(\begin{array}{c} v_{1}\\ v_{2} \end{array}\right)=\left(\begin{array}{cc} \mu_{1} & \mu_{2}\\ \rho_{e,1} & \rho_{e,2} \end{array}\right)\;\left(\begin{array}{c} v_{1}\\ v_{2} \end{array}\right).$$

To obtain the material fractions, the vector of the obtained measurements (μ,ρ) is multiplied with the inverse change of basis matrix $A^{-1}$.

In Sections 2.2 and 2.3, a material decomposition was performed using soft tissue and lead as basis materials. Their respective X‐ray interaction properties are provided at the end of Section 4.3. Because in Section 2.3 the attenuation value of native tissue at 20keV was unknown, it was estimated using the lookup‐table entry for water ($\mu_{w}=0.81$). Note that due to variations in the density of soft tissue, volume conservation (i.e. $v_{1}+v_{2}=1$) does not necessarily have to be fulfilled [42]. The volume fraction can be converted into a molar concentration

(17) $$c=v\frac{\rho }{M},$$

provided the molar mass M and mass density ρ of the basis material are known.

In the Supporting Information, we provide a robustness test demonstrating that the two‐material decomposition approach accurately recovers stain concentrations (relative errors <1.8%) across different soft tissue types, with tissue heterogeneity manifesting primarily in the retrieved soft tissue fraction rather than introducing systematic bias in the quantified stain concentration.

### 4.6 K‐Edge Subtraction Imaging

To provide validation of the modulation‐based virtual histology method described in this paper, K‐edge imaging of the same sample was performed. This approach can be used to retrieve the distribution of a target element by use of its K‐edge, a sudden increase in X‐ray absorption that occurs when the energy of the beam becomes sufficiently large to overcome the binding energy of the innermost electron shell of a material. An estimate of the volume fraction v of a target material X may be recovered by taking two complementary absorption tomography scans ($\mu_{1},\mu_{2}$) at energies $E_{K}-\Delta E$ and $E_{K}+\Delta E$, where $E_{K}$ is the energy of the K‐edge of the target material and ΔE is some small energy increment.

The attenuation in each voxel is attributable to a sum of the attenuation of the target material X and all other materials, here referred to as the background B [87]:

(18) $$\mu_{i}=v\;\mu_{X}\left(E_{K}\pm \Delta E\right)+\left(1-v\right)\;\mu_{B}\left(E_{K}\pm \Delta E\right).$$

Since any K‐edges related to the background material are not within the energy scan range, which was chosen to cover the K‐edge of the target material, we assume

(19) $$\mu_{B}\left(E_{K}+\Delta E\right)\approx \mu_{B}\left(E_{K}-\Delta E\right),$$

allowing us to retrieve the approximate volume fraction of the target material using

(20) $$v_{X}=\frac{\mu_{2}-\mu_{1}}{\mu_{X}\left(E_{K}+\Delta E\right)-\mu_{X}\left(E_{K}-\Delta E\right)}.$$

### 4.7 Comparison to Optical Histology

The histological slice was digitized using the ZEISS Axio Scan.Z1 microscope (*Carl Zeiss AG*, Oberkochen, Germany) in conjunction with a 20‐fold magnification objective. The image was converted to greyscale by computing the average of all three color channels and applying a percentile normalization in the range of 1% to 99%. Using the 2D‐3D registration algorithm described in Ref. [19], the 4 closest matching slices through the 3D concentration volume were identified and deformably registered. Since a single slice in the reconstructed volume has a thickness of 1.28μm, the slice depicted in Figure 5b shows a mean image of the 4 slices to match the slice thickness of conventional histology, which was 5μm. In addition, an unsharp mask as implemented in the *scikit* python library [88] (radius 1, strength 3) was applied to the image in panel (b).

The resolution in the virtual histology image was determined in the axial plane by performing a Fourier ring correlation [89] in the implementation described in Ref. [78]. The calculation was repeated for 100 consecutive slices, both using the half‐bit and more conservative full‐bit criterion, yielding the mean values stated in Section 2.3.

### 4.8 Offset Rotation‐Axis Scanning

A detector's horizontal field of view can be extended by up to a factor of 2 using offset rotation‐axis scans. In this measurement protocol, the sample's rotation axis is offset such that its center lies closer to one edge of the detector (instead of the middle). The sample then needs to be imaged over a range of 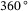 instead of 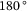. While the procedure is successfully used for propagation‐based synchrotron imaging [90, 91], its application to modulation‐based imaging data requires some additional image processing described in the following.

After phase‐retrieval with UMPA, each differential phase projection $\phi_{x}\left(x,y\right)$ and $\phi_{y}\left(x,y\right)$ is stitched with the projection recorded from the opposite angle (i.e., 180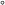 apart), which is horizontally mirrored. In the case of $\phi_{x}\left(x,y\right)$, the sign of the mirrored projection needs to be flipped. As this process leads to redundant information in the overlap region, a linear blending is applied to provide a smooth transition – as employed in Ref. [92]. The resulting differential phase projection of the full field of view is calculated using

(21) $$\begin{array}{cc} \phi_{i}\left(x,y\right) & =\alpha \left(x\right)\;\phi_{i,f}\left(x,y\right)\\ & \;+\left(1-\alpha \left(x\right)\right)\;\phi_{i,b}\left(x,y\right), \end{array}$$

where i refers to either the differential phase signal in the x‐ or y‐direction and $\phi_{i,b}$ are two differential phase projections spaced 180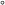 apart. The linear blending function is defined as

(22) $$\alpha \left(x\right)=\left\{\begin{array}{cc} 1 & x<x_{\mathrm{start}}\\ 1-\frac{x-x_{\text{start}}}{\Delta w} & x_{\mathrm{start}}\leq x\leq x_{\mathrm{start}}+\Delta w\\ 0 & x>x_{\mathrm{start}}+\Delta w. \end{array}\right.$$

Here, Δw is the width of the overlap region and $x_{\text{start}}$ is the x‐coordinate corresponding to the start of the overlap. The transformation of the differential projections into electron densities then proceeds as previously described in Section 4.4.

## Author Contributions

D.J., J.He., and K.S.M. conceptualized the experiments. D.J., D.M.P., M.‐C.Z., J.He. and K.S.M. developed the analysis methods. D.J., M.‐C.Z., J.B.T., K.S.M., J.A., and S.J.A. prepared and performed the MCT experiment. L.M.P., P.I., S.B., and M.B. prepared the samples and optimized the protocols. J.C. performed the deformable registrations and matched the histology slice. L.M.P., P.I., J.A., and S.W. assisted with data analysis. D.J., F.B. and J.M. performed the experiment at P07. D.J. and J.U.H. performed the P05 experiment. All authors reviewed the manuscript.

## Conflicts of Interest

J.C. is an employee of ImFusion GmbH. All other authors declare no competing interests.

## Supporting information


**Supporting File**: advs73787‐sup‐0001‐SuppMat.pdf.

## Data Availability

The data that support the findings of this study are available from the corresponding author upon reasonable request.
