# Supplementary material for: Quantitative Stain Mapping in X‐Ray Virtual Histology
Source: Adv Sci (Weinh). 2026 Jan 20;13(21):e19783. doi: 10.1002/advs.202519783 (PMC13073281; doi:10.1002/advs.202519783)
Supplement: Supplementary file 1 — Supporting File: advs73787‐sup‐0001‐SuppMat.pdf. [file ADVS-13-e19783-s001.pdf]

Supporting Information

Quantitative Stain Mapping in X-ray Virtual Histology

Dominik John, David M. Paganin, Marie-Christine Zdora, Lisa Marie Petzold, Patrick Ilg, Junan Chen, Sara Baggio, Johannes B. Thalhammer, Sami Wirtensohn, Julian Moosmann, Jörg U. Hammel, Felix Beckmann, Samantha J. Alloo, Jannis N. Ahlers, Madleen Busse, Julia Herzen, Kaye S. Morgan*

Robustness of Material Decomposition to Tissue Heterogeneity

To assess the robustness of the two-material decomposition approach in the presence of tissue heterogeneity, we provide a numerical validation using three tabulated human tissue compositions from the ICRP database [1] here. While these specific tissues (skeletal muscle, adipose tissue, and skin) are not present in the sample examined in our study, they represent a realistic range of soft tissues that may be found within the same histological sample and serve as a test case for the decomposition method's ability to accurately retrieve stain concentrations despite variations in the underlying tissue composition.

For the robustness analysis, we calculate the electron density and linear attenuation coefficient at 25 keV for the three soft tissues using the *xraylib* library [2] (Table 1). We define the baseline soft tissue material for decomposition as the arithmetic mean of these three tissue types. This is equivalent to the result one would obtain by measuring an unstained reference sample and calculating its average properties, as proposed in this study. We then simulate samples consisting of each pure tissue type mixed with 1% lead by volume and perform material decomposition using the averaged soft tissue and lead as basis materials.

Table 1. Material properties of pure soft tissues and their average. Electron densities and linear attenuation coefficients were calculated at 25 keV using elemental compositions from ICRP Publication 89 [1].

**Material**	**ρ_e (nm^{-3})**	**μ (cm^{-1})**
Skeletal muscle	343.9	0.610
Adipose tissue	309.3	0.381
Skin	363.7	0.584
**Avg. soft tissue**	**339.0**	**0.525**

Table 2 summarizes the retrieved stain concentrations from material decomposition. The decomposition successfully recovers the 1% lead concentration with relative errors below 1.8% across all tissue types. Notably, variations in the underlying tissue composition manifest primarily as changes in the retrieved soft tissue fraction rather than systematic bias in the stain concentration. This demonstrates that the two-material decomposition approach remains accurate for stain quantification even when the actual tissue composition deviates from the averaged baseline, supporting the validity of this method for heterogeneous biological samples. Deviations in the retrieved stain concentration would likely increase considerably for tissues with substantially different X-ray interaction properties, such as mineralized bone.

Table 2. Retrieved fractions from decomposition of simulated tissue-stain mixtures. Each tissue was mixed with 1.00% lead by volume and decomposed using averaged soft tissue and lead as basis materials. The error represents the relative error in the retrieved stain concentration.

Tissue mixture	Avg. soft tissue (v/v, %)	Lead (v/v, %)	Error (%)
Skeletal muscle + 1% lead	100.3	1.01	1.40
Adipose tissue + 1% lead	90.5	0.98	1.78
Skin + 1% lead	106.2	1.00	0.38

References

[1] Valentin, J. (2002). Basic anatomical and physiological data for use in radiological protection: reference values: ICRP Publication 89: Approved by the Commission in September 2001. *Annals of the ICRP*, 32(3-4), 1-277.
[2] Schoonjans, T., et al. (2011). The xraylib library for X-ray–matter interactions. Recent developments. *Spectrochimica Acta Part B: Atomic Spectroscopy*, 66(11-12), 776-784.
